# Quantificational 4D Visualization of Industrial Electrodeposition

**DOI:** 10.1002/advs.202101373

**Published:** 2021-10-28

**Authors:** Handong Jiao, Zhaoliang Qu, Shuqiang Jiao, Yang Gao, Shijie Li, Wei‐Li Song, Mingyong Wang, Haosen Chen, Daining Fang

**Affiliations:** ^1^ Institute of Advanced Structure Technology Beijing Institute of Technology Beijing 100081 P. R. China; ^2^ State Key Laboratory of Advanced Metallurgy University of Science and Technology Beijing Beijing 100083 P. R. China

**Keywords:** industrial electrodeposition, in situ visualization, quantificational monitor, X‐ray tomography technology

## Abstract

Electrodeposition is a fundamental technology in modern society and has been widely used in metal plating and extraction, etc. However, extreme reaction conditions, including wide operation temperature ranges and corrosive media (molten salt/oxide systems as a particular example), inhibit direct in situ observation of the electrodeposition process. To visualize the electrode kinetics in such “black box,” X‐ray tomography is employed to monitor the electrochemical processes and three‐dimensional (3D) evolution of morphology. Benefiting from the excellent penetration of X‐ray, a non‐destructive and non‐contact in situ four‐dimensional (4D) visualization of Ti deposition is realized. Real‐time 3D reconstructed images reveal that the counterintuitive nucleation and growth process of a mesoscale Ti dendrite at both solid and liquid cathodes. According to 3D morphology evolution, unusual mechanism based on synergetic effect of the diffusion of metallic Ti and local field enhancement is achieved utilizing a simulation method based on a finite element method. This approach allows for timely and accurately regulating the electrodeposition process upon in situ monitored parameters. More importantly, the 4D technique upon operando X‐ray tomography and numerical simulation can be easily applied to other electrodeposition systems, which will help deeply understand the internal kinetics and the precise optimization of the electrodeposition conditions.

## Graphical abstract

Quantificational 4D visualization of the electrodeposition technology under extreme conditions based on X‐ray tomography technique is developed, which can be used to discover the unprecedent electrode process in real time from a visible state. It is also helpful to fundamentally understand the electrode kinetics, accurately control the process of the industrial electrodeposition, and achieve high‐quality products.

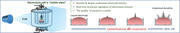

## 1 Introduction

Industrial electrodeposition^[^ ^1^ ^]^ is a well‐established technology in the field of electrochemical engineering, particularly in surface engineering and electrometallurgy,^[^ ^2^, ^3^ ^]^ because it is more controllable for the extraction and plating of metals and alloys.^[^ ^4^, ^5^, ^7^, ^8^, ^9^, ^10^, ^11^ ^]^ In general, the quality of electrodeposits, including coating uniformity, favorable brightness and adhesion, etc., is mainly affected by the operating parameters, such as, current density, temperature, concentration, and local morphology of electrodes.^[^ ^11^ ^]^ To understand in‐depth relationship between operational parameters and coating properties, it is important to employ appropriate characterization techniques and analysis methods. Unfortunately, the related mechanism in the industrial electrodeposition processes has not been well clarified, owing to the lack of quantitative method for in situ observation and analysis.

**Figure 1:**
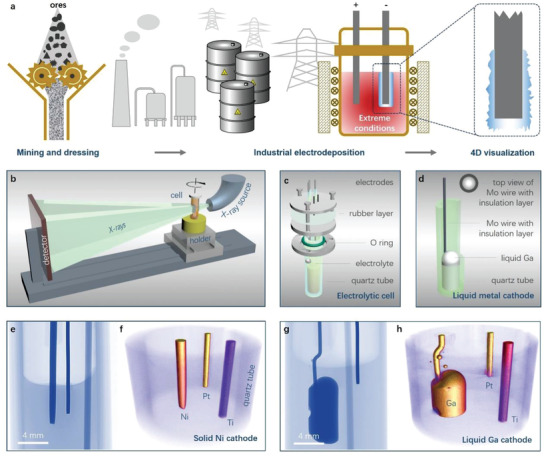
Schematic of 4D visualization setup for industrial electrodeposition. a) A typical and simplified industrial electrodeposition process under extreme conditions (high temperature or strongly corrosive environment). b) 3D representation of the assembled 4D characterization system. c,d) 3D‐views of the electrolysis cell and the liquid Ga electrode. e,f) 2D vertical‐section image and 3D rendering image of the electrolysis cell with a solid Ni working electrode. g,h) 2D vertical‐section image and 3D rendering image of the electrolysis cell with a liquid Ga working electrode.

These challenges are mainly linked with the extreme electrodeposition conditions as well as the so‐called “black box” reaction system, resulting in promoting the difficulty of establishing equipment and methodology to quantitatively analyze the processes timely (**Figure** **1a**). For instance, the operation temperature of the molten salts^[^ ^12^ ^]^ and oxides^[^ ^14^, ^15^ ^]^ electrolytes is in the broad range of 200–1600 °C. In this case, there is a lack of operando techniques for monitoring the electrodeposition behaviors under high‐temperature conditions, even more severe with the presence of thermal radiation and corrosive environment.^[^ ^5^, ^16^ ^]^ Moreover, the thermal insulation materials and chambers of electrolytic cells prevents the signal from penetrating through the reaction chamber.^[^ ^9^ ^]^ In the attempts of tackling the challenges, up to date, the combination of traditional electroanalytical methods coupling with ex situ characterization techniques is developed as the compromised approach, while the real‐time local information of the actual electrode evolution upon kinetics is still insufficient.

In a pioneering exploration, Walsh et al. introduced X‐ray techniques in such “black box” and obtained internal information on electrodeposition, which opens a new approach to the study of electrodeposition processes.^[^ ^17^, ^18^ ^]^ Subsequently, various studies based on ex situ and in situ X‐ray techniques were employed in the electrochemistry. For instance, Young Taek Jeek and coworkers^[^ ^18^ ^]^ investigated microstructure evolution of the electrodeposits during electroreduction processes by using ex situ X‐ray computed tomography. On the other hand, other researchers recently used in situ X‐ray techniques to demonstrate the dynamic processes of electrodes in the energy industry.^[^ ^20^, ^21^, ^22^ ^]^

Aiming to operando monitoring and analyzing the electrodeposition process, in the present contribution, we employ an X‐ray tomography technique to open the “black box” via establishing the four‐dimensional (4D) visualization methodology (in situ evolution of three‐dimensional (3D) spatial imaging with time), aiming to operando monitor and analyze the electrodeposition process. With the unique advantages on sufficient penetration capacity^[^ ^23^, ^24^, ^25^ ^]^ and non‐destructive and non‐contact features, the X‐ray tomography allows for reconstructing the growth process of the deposited metal dendrites timely. With the assistant of the numerical method, we could extract the quantitative results including geometry size of electrodeposits using X‐ray tomography (at the spatial resolution of 6–10 µm). These results are significant for analyzing current efficiency and coating quality at different electrolysis stages. To verify the validity of such 4D visualization methodology, Ti electrodeposition was carried out using both solid Ni cathode and liquid Ga cathode in a corrosive ionic liquid electrolyte, followed by in situ monitoring and reconstructing the Ti dendrites and cathode morphology. Because electrochemical strategies are considered as the promising technologies for a low‐cost and short‐process production approach to obtain metallic titanium in comparison with the traditional method of Kroll process, Ti electrodeposition was selected as the demonstration in this work.^[^ ^5^ ^]^ In our previous studies of past decades, significant efforts have been drawn to develop electrodeposition techniques for Ti extraction and purification.^[^ ^26^, ^27^, ^28^ ^]^ On the other hand, liquid metal cathodes were commonly used for the underpotential deposition (UPD) of Ti during molten salt/oxide electrolysis. In this process, we have successfully extracted titanium and produced titanium alloys in the above high‐temperature system.^[^ ^28^ ^]^ In the ionic liquid electrolyte, Ga (liquid state at room temperature) was used as the liquid cathode, with expectation of underpotentially depositing Ti on the Ga cathode. Additionally, multi‐physical modeling and simulation was also introduced to understand the mechanism of the observed unusual dendrite evolution in the electrode process. Apparently, the results of 4D visualization and analysis via experimental and simulating methods here have created a stage for well understanding the critical issues in the electrodeposition process under extreme high‐temperature molten salt or even molten oxide systems. Whereby, the high‐yield products with premium quality from industrial electrodeposition would be expected.

## 2 Results

Figure 1b depicts the schematic of the as‐designed integrated configuration for operando visualization and quantitative measurement. For meeting the testing requirement of 4D X‐ray tomography, a specific size of electrolytic cell (Figure 1c and Figure S1d,e, Supporting Information) is fixed vertically to the rotation holder of X‐ray system, followed by connecting with an electrochemical test system. More photographs on direct in situ system are given in Figure S1a–c, Supporting Information, showing that the electrolytic cell could be well integrated with the X‐ray tomography system. As a demonstration, electrode kinetic processes of metallic Ti electrodeposition on both solid Ni and liquid Ga electrodes were studied (Figure 1d and Figure S1f, Supporting Information) in a corrosive ionic liquid electrolyte. Figure 1e,g show the 2D vertical cross‐section images of the electrolytic cells with different working electrodes of Ni and Ga, which were extracted from the X‐ray scan data. The corresponding 3D images and video are exhibited in Figure 1f,g, as well as Movie S1, Supporting Information. Noted that there are some small balls above the liquid Ga cathode which are metallic Ga drops adhered on the surface of the cathode system. Because of the density difference between the metallic electrodes and ionic liquid electrolytes, X‐ray tomography can distinguish the electrodes from the electrolyte simply, and a precise internal structure of the electrolytic cell could be clearly shown in both 2D section images and 3D rendering images. These findings primarily validate the stability and reliability of the integrated operando measurement system.

**Figure 2:**
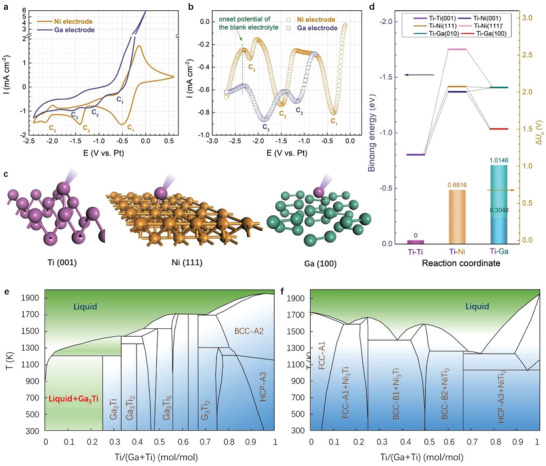
Electrochemistry and DFT calculation of Ti electrodeposition. a) Cyclic voltammograms and b) square wave voltammograms of Ti on Ni and Ga electrodes. Scan rate: 10 mV s^−1^, frequency: 10 Hz. c) Schematic illustration of Ti electroreduction on different metallic substrates in an atomic‐scale. d) Calculated the binding energy diagram of Ti at three metallic substrates (denoted by segment plot) and the underpotential shift (Δ*U*p, calculated by C_i_‐C_3_ (Ni)) achieved from the CVs (denoted by column plot). e,f) Ti‐Ga and Ti‐Ni phase diagrams.

Subsequently, electroanalytical measurements were performed in the above three‐electrode configuration to obtain the essential parameters, which would be used for the subsequent in situ electrodeposition and understanding electrochemical reaction at working electrodes. Initially, to avoid the electrochemical decomposition of the solvent electrolyte ([BMIm]BTA) without the solute of TiCl_4_, polarization test was carried out to confirm the stable electrochemical windows. This electrolyte was selected as the room‐temperature ionic liquid electrolyte, and could be used to produce purity titanium metals, according to the previous studies. Two cathodic polarization curves demonstrate that the onset potential of [BMIm]BTA electroreduction was around −2.4 V versus Pt Quasi‐RE with the presence of both Ni and Ga electrodes (Figure S3a,c, Supporting Information). However, the onset potential of oxidation was different at two electrodes (Figure S3b,d, Supporting Information), which was mainly resulted from the oxidation of metallic electrode.^[^ ^30^, ^31^ ^]^ Within the stable electrochemical window, cyclic voltammetry (CV) was carried out under different scan rates. As shown in **Figure** **2a**, there were three cathodic peaks, assigned as C_1_ (Ni), C_2_ (Ni), and C_3_ (Ni), and the corresponding anodic peaks of Ni electrode could be denoted by yellow line, which was also confirmed by square wave voltammetry (SWV) (yellow circle in Figure 2b). According to the study by Mukhopadhyay et al., the cathodic peaks C_1_ and C_3_ corresponded to the reduction processes of Ti^4+^→Ti^3+^ and Ti^3+^→pure Ti, respectively,^[^ ^31^ ^]^ which indicates pure metallic Ti deposited on the Ni electrode at the potential of C_3_ (Ni) (i.e., about −2.2 V vs Pt Quasi‐RE). At the cathodic peak C_2_ (Ni), a possible process is the UPD of Ti atoms on the Ni electrode because the UPD in the electrodeposition process is a very common phenomenon at the active electrodes, specifically at Ni, Fe, and liquid metals, etc.^[^ ^30^, ^33^, ^34^, ^35^ ^]^ This process could be described as Ti^3+^→Ti‐Ni alloys, which also demonstrated an alloying process.^[^ ^36^, ^37^ ^]^

On the other hand, the cathodic process of Ti on the Ga electrode was also revealed by CV (violet line in Figure 2a). Three cathodic peaks, assigned as C_1_ (Ga) C_2_ (Ga) and C_3_ (Ga), were also detected on the Ga electrode and the potentials of C_1_ (Ga) and C_1_ (Ni) were very close. However, compared with the potential of the C_2_ (Ni) and C_3_ (Ni), the CV of Ga shifted the potentials of C_2_ (Ga) and C_3_ (Ga) to the positive values. The potentials of the C_2_ (Ga) and C_3_ (Ga) were also studied by SWV (violet circle in Figure 2b), which further confirmed the results of CV. It is noteworthy that C_1_ (Ga) does not show in SWV because the SWV started from the OCP (about −0.8 V vs Pt Quasi‐RE), which is the negative potential region of the C1. According to Nernst equation, the reduction potential of Ti^3+^→pure Ti should be the same on either Ni or Ga electrode under the same condition. Consequently, the difference between C_3_ (Ga) and C_3_ (Ni) indicates that C_3_ (Ga) represents the UPD of Ti on Ga electrodes rather than preparation of pure metallic Ti, which could be also applied to C_2_ (Ga). In brief, both C_3_ (Ga) and C_2_ (Ga) could be described as Ti^3+^→Ti‐Ga alloys. Meanwhile, two UPD or alloying processes obtained on the Ga electrode may be potentially linked with the Ti‐Ga phase diagram, in which a mixture liquid phase appears at room temperature (Figure 2e). Apparently, this observation is different from the Ti‐Ni phase diagram (Figure 2f).

Furthermore, first‐principles calculation based on density functional theory (DFT) was carried out to understand the essential mechanism of the UPD in this work, which is also a necessary basis for understanding the following kinetic process. Note that the fundamental and theory of UPD has been extensively discussed previously.^[^ ^37^ ^]^ A recent study showed that the underpotential shift (Δ*U*p, calculated by C*_i_* (X)‐C_3_ (Ni), *i* = 1 and 2, X = Ni and Ga) was directly proportional to the binging energy (*G*_BE_) between the deposited atom and the substrate atom.^[^ ^38^ ^]^ Based on this finding, a comprehensive theoretical calculation was conducted, and various crystal planes and active sites of the substrate metals were considered. Therefore, a series of binding energies of Ti‐Ti, Ti‐Ni, and Ti‐Ga could be obtained. The calculated *G*_BE_ and Δ*U*p are summarized in Figure 2d, which indicates that the *G*_BE_ and Δ*U*p were linearly dependent in the Ti‐Ni system. However, partial *G*_BE_ and Δ*U*p were linearly dependent in the Ti‐Ga system. Such unique phenomenon shown in Ti‐Ga system would be resulted from the enhanced vibratility, long‐range disorder nature and solubility of liquid Ga atom. As aforementioned, there is a liquid phase region in the Ti‐Ga phase diagram at room temperature. This enables the dissolution of metallic Ti atom into Ga electrode and exposing of the fresh long‐range disordered Ga atom at interface, which eventually makes the electroreduction process of Ti on the Ga electrode more complicated. Although it is difficult to observe this kinetic process directly by experimental approach, we captured certain evidences from the extended CV measurements. As shown in Figure S4d, Supporting Information, the polarization value (Δ*U*, defined in Figure S4a,d, Supporting Information) obtained on the Ga electrode was higher than that on the Ni electrode, which corresponds to the kinetic nature of the diffusion of Ti atom into Ga electrode. According to these findings, the UPD of Ti atom on the solid Ni and liquid Ga substrates can be schematically illustrated in Figure S4e,f, Supporting Information. During the UPD, Ti ions are electro‐reduced, which subsequently leads to the formation of Ti—Ni bonding and a thermodynamically stable Ti monoatomic layer.^[^ ^37^ ^]^ Due to the vibration and disordered nature of liquid Ga atom, on the contrary, the Ti monoatomic layer on the Ga surface is thermodynamically unstable. This would result in the diffusion of Ti atom into Ga electrode, which also agrees with the self‐healing and dendrite inhibition properties of liquid metal electrodes in the current literature.^[^ ^39^ ^]^

**Figure 3:**
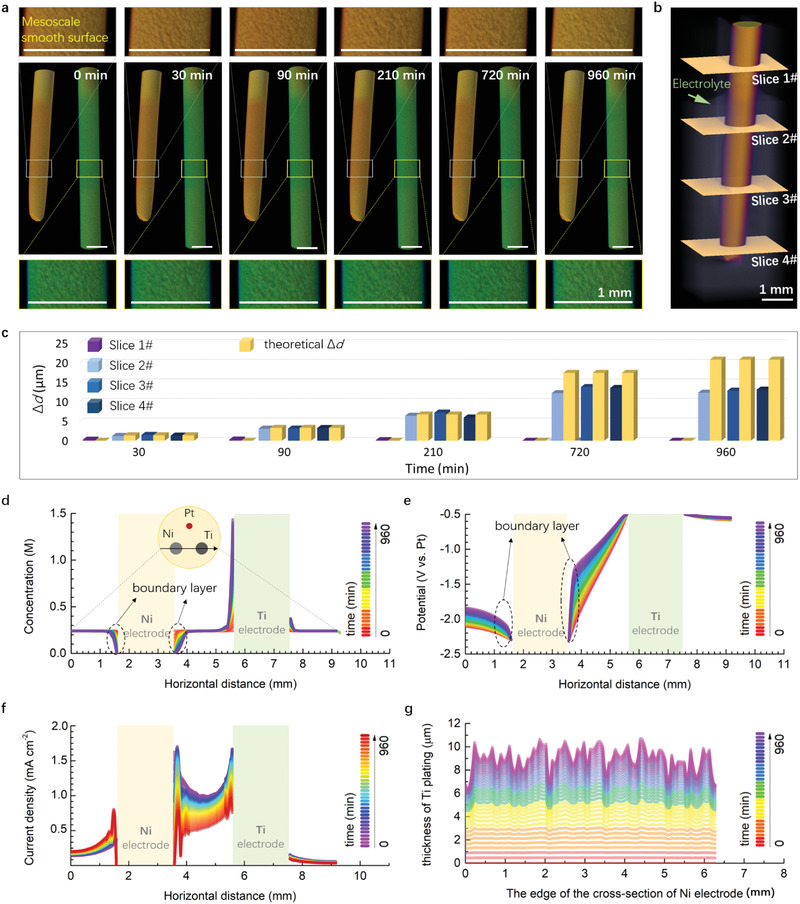
4D visualization of Ti electrodeposition on the solid Ni. a) 3D reconstructed images of the Ni working electrode and Ti counter electrode at different electrolysis time. b) The demonstration for the extraction of cross‐section slice. c) The change of the Ti plating thickness on the Ni electrode. Data are presented as mean (*n* = 3). The evolution, following the black arrowed line, of d) TiCl4 concentration, e) electric filed, and f) current density with time achieved by simulations. g) The change of the thickness of Ti plating on the edge of the cross‐section of Ni electrode calculated from the simulation results.

Subsequently, 4D visualization of the electrodeposition process was performed via X‐ray tomography technique, according to the methods described in the Experimental section. The current‐time profile of the 4D electrodeposition of metallic Ti on the Ni electrode is shown in Figure S5, Supporting Information. Accordingly, the profile dropped rapidly at the initial state and then decreased slowly, corresponding to the nucleation and growth process of metallic Ti. Because the area of the Ni electrode is highly low, the Ti product was characterized by scanning electron microscopy (SEM) and EDS. As shown in Figure S6b,c, Supporting Information, Ti atom was evenly distributed on the surface of the Ni electrode, which indicates that metallic Ti could be electrodeposited on the Ni electrode in the TiCl_4_‐[BMIm]BTA ionic liquid, consistent with the published results.^[^ ^31^ ^]^ **Figure** **3a** depicts the in situ reconstructed 3D images of the Ni working electrode and Ti counter electrodes at different electrolysis time. The results show that there is no obvious morphological change over electrolysis time at both Ni and Ti electrodes, and the corresponding smooth surface appeared under the spatial resolution of 11 µm using the X‐ray tomography in this work. Note that the scale discussed here is defined by the spatial resolution of the X‐ray tomography, suggesting the mesoscale recognized in this study. This finding illustrates that it is difficult for metallic Ti grains to evolve into a mesoscale Ti dendrite on the Ni electrode. Furthermore, the average thickness (Δ*d*) of Ti plating was calculated by a statistical method (Figure S7a,b, Supporting Information), and the corresponding results are summarized in Figure 3c. During electrodeposition, Δ*d* increased while the gap between the theoretical and experimental values increased, which indicates the current efficiency decreased with time. In principle, there are many factors that can reduce the current efficiency in electrodeposition. Nevertheless, the dominant one is the disproportionated reaction of the transition state in Ti electrodeposition systems, because Ti is a multivalent element and its reduction is normally a multi‐step process.^[^ ^30^, ^32^, ^33^, ^34^ ^]^ As a comparative study, on the other hand, the microscale morphologies of the Ni working electrode and Ti counter electrode after electrolysis for 960 min were characterized by SEM. Compared with the original surfaces of Ni and Ti (Figure S8a,b, Supporting Information), the treated surfaces on both Ni and Ti displayed rough morphologies (i.e., roughness about 2–3 µm). Evidence based on the X‐ray tomography and SEM images confirm that Ti plating on the Ni electrode is smooth on the mesoscale while it is rough on the microscale (Figure S8c, Supporting Information). Although some microscale morphology results were missed due to the mesoscale spatial resolution in this work, the aforementioned observation supports the valuable information of 4D visualization from Ti electrodeposition. The time‐dependence changes on the volume of product and current efficiency were also detected by this method.

To understand the 4D visualization of Ti electrodeposition, the modelling and simulation of the electrode processes were performed on COMSOL Multiphysics software, aiming to exhibit 3D evolution of concentration field, electric field, current density, and plating thickness during electrolysis. Figure 3d shows the evolution of TiCl_4_ concentration during electrolysis, and the extended 3D evolution results are provided in Figure S9 and Movie S2, Supporting Information. Due to the slow diffusion and consumption of Ti ions via electrodeposition, the concentration near the Ni electrode was smaller than that in bulk electrolyte, resulting in the generation of the typical boundary layer (BL). Likewise, the BL was also formed in the local region of the Ti electrode, while the concentration within the local regions exceeded that in the bulk electrolyte, indicating the electro‐dissolution of Ti electrode. Moreover, the thickness of BL and the concentration gradient in the BL increased dramatically with the electrodeposition time. The corresponding evolution of the ohmic potential drop and current density drop near the Ni electrode were also clearly visible in Figure 3e,f, Figures S10 and S11, Supporting Information (also in Movies S3 and S4, Supporting Information). The structure of the BL (circled by a black dotted line in the Figure 3d) was variable in the different regions of the electrodes, which is mainly generated by the inhomogeneous distribution of electric filed (Figure S10b, Supporting Information) and current density (Figure S11, Supporting Information). The thickness evolution of the Ti plating was also quantitatively calculated. As shown in Figure 3g, Figure S12 and Movie S5, Supporting Information, the thickness monotonically increased with time, and the roughness caused by the inhomogeneous distribution of fields increased as well. However, the roughness always was maintained in a microscale (i.e., about 2–4 µm), which well matches our experimental results of both the X‐ray tomography and the SEM.

**Figure 4:**
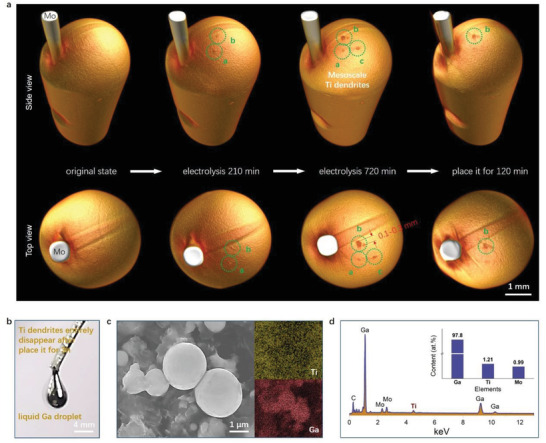
4D visualization of the unprecedent Ti dendrites on liquid Ga. a) Time‐dependence 3D reconstructed images of the Ga working electrode at different electrolysis time. b) The photograph and c) SEM image of the liquid Ga after electrolysis 720 min and hold 120 min. d) EDS result of the Ga working electrode with Ti atom.

In the electrometallurgy, it is noticeable that liquid metal electrodes attracted increasing attention because of their outstanding merits, such as selective reduction,^[^ ^40^ ^]^ UPD, and dendrite inhibition, etc., compared with solid electrodes. Subsequently, we employed liquid Ga as electrode for studying the kinetic process of Ti electrodeposition. Figure S13, Supporting Information, shows the current‐time profile of Ti electrodeposition on Ga electrode. The profile decreased immediately at the initial stage, and then remained stable, demonstrating a good stability of the interface between the liquid Ga and electrolyte.^[^ ^15^, ^30^, ^41^ ^]^ 3D reconstructed images of the Ga working electrode at different electrolysis time are given in **Figure** **4a**, where three distinct mesoscale Ti dendrites (circled by green dotted line and numbered a, b, and c) were tracked by X‐ray tomography, which is different from the mesoscale smooth surface of the Ni. Obviously, such unusual phenomenon is rarely found in the conventional results, specifically in the case that the mass of the deposited Ti is far less than that of the liquid Ga electrode. Interestingly, it is noted that this observation is difficult to track according to Ti‐Ga phase diagram. As shown in the photograph of Figure 4b and SEM image of Figure 4c as well as Figure S14, Supporting Information, the Ga electrode after electrolysis turned back into liquid state when it was collected from the electrolytic cell. Therefore, the time‐dependence 4D visualization of Ti dendrites on the liquid electrode in this study could not be well found by traditional ex situ characterizations.

In the first stage within electrolysis for 210 min, two Ti dendrite seeds (a and b spots in Figure 4a) appeared. Upon electrolysis for 720 min, these two Ti dendrite seeds grew up, and meanwhile the third Ti dendrite (c spot in Figure 4a) appeared. The coverage rate of Ti dendrites, the area ratio of the Ti dendeites and the liquid Ga cathode, increased from 0.052% at 120 min to 0.628% at 720 min. Although the coverage rate is low, its increasing rate is not to be overlooked. We can expect that the liquid Ga cathode could be totally covered by the solid Ti dendrites after a long‐term uncontrolable electrolysis, which is harmful for the industrial application of electrodeposition. With settling for 120 min, two small Ti dendrites (a and c spots in Figure 4a) disappeared and the size of the larger Ti dendrite (spot b in Figure 4a) decreased after settling the electrolytic cell for 120 min. Figure 4d shows the EDS results after the Ga electrolysis for 720 min, and Ti was detected in the product. The results indicate that it is feasible for using the liquid Ga to electrodeposit Ti, which should be linked with the Ti dendrite observed upon electrolysis.

**Figure 5:**
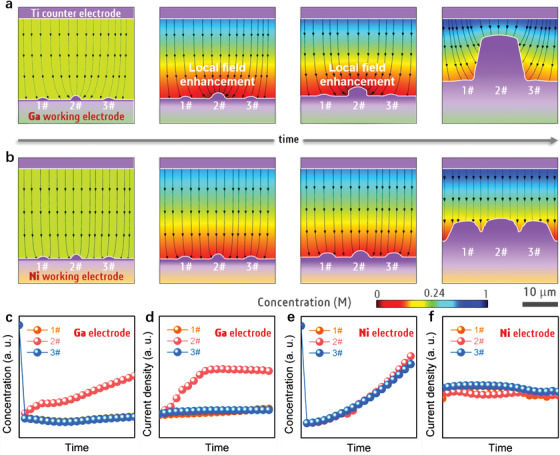
Understanding of the unprecedent Ti dendrite. Simulations morphological evolution of a) the mesoscale Ti dendrite on liquid Ga surface, namely “growth model of diffusion‐induced local field enhancement” and b) the microscale Ti dendrite on Ni surface during electrolysis. The current density is clearly shown by the black line with arrow. c,d) The plots of the local concentration and local current density near 1#, 2#, and 3# Ti particles at Ni electrode with time, obtained from (a). e,f) The plots of the local concentration and local current density near 1#, 2#, and 3# Ti particles at Ga electrode with time, obtained from (b).

Subsequently, simulation was carried out to clarify the mechanisms of the unusual phenomenon on the liquid Ga electrode. For comparing the electrolysis processes and conditions, the effects of both solid Ni and liquid Ga on the Ti electrodeposition were analyzed. For the typical electrode kinetics, the Ti ions would be converted into Ti atoms via electro‐reduction process in the initial stage, followed by diffusing into the solid/liquid cathodes. Hence, the initial geometric model along with corresponding parameters and boundary conditions was established to simulate Ti electrodeposition on both Ni and Ga. Meanwhile, it is assumed that three microscale Ti nucleus were modelled in the original geometric models on both Ni and Ga electrodes, according to the practical electro‐crystallization kinetics. In the parameter setting, the initial diffusion coefficients in the solid Ni and liquid Ga were set on the orders of 1 × 10^−15^ m^2^ s^−1^ and 1 × 10^−10^ cm^2^ s^−1^, respectively.^[^ ^41^ ^]^ The simulation results (**Figure** **5**) well agree with our experimental findings, which verifies the hypothesis that the fast diffusion of Ti atoms into liquid Ga electrode would induce the Ti dendrites. Because of different dendrite size, the larger dendrite (2#) would be largely maintained on the surface of Ga electrode, while the smaller ones (1# and 3#) would be dissolved. In the subsequent electrodeposition, the larger dendrite could be induced concentrated current densities (Figure 5c), which is favorable for dendrite growth within higher local ion concentration in the BL (Figure 5d). This phenomenon could be understood as the “Matthew Effect,” and could be described as a “growth model of diffusion‐induced local field enhancement.” On the Ni cathode (Figure 5b), on the contrary, the diffusion coefficient is much lower than that in the solid Ni, and Ti nucleus could be well maintained on the solid electrode surface. Thus, the local filed enhancement (Figure 5e,f) would appear near all the local region of the Ti nucleus (1#, 2#, and 3#), and all the nucleus would grow into mossy dendrites in the subsequent electrodeposition.

## 3 Discussion

**Figure 6:**
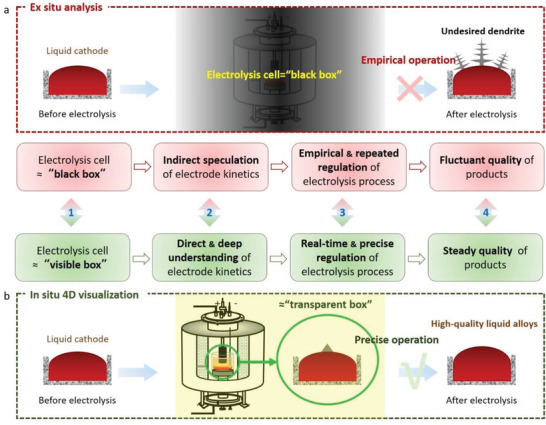
Perspective of the quantificational 4D visualization analysis based on in situ CT technique. a) Traditional ex situ anaylsis: Empirical operation and process optimization owing to the experiments from “black box” electrolysis cell. b) Novel analysis upon in situ 4D visualization: Quantificational 4D visualization analysis platform for precisely manipulating the electrolysis process to achieve high‐quality products.

**Figure** **6** illustrates the advantages and contribution of such 4D visualization technology with comparison between the traditional ex situ analysis system and the quantificational 4D visualization analysis platform. Generally, the internal status of the opaque electrolysis cells could not be well analyzed via traditional ex situ characterization methods, typically with the presence of electrolyte under extreme conditions (high temperature, corrosion, etc.). As a consequence, the electrolysis process could not be real‐time regulated and the process optimization is usually based on empirical methods. Thus, a series of repeated experiments are required to be made for obtaining the strategy to improve electrolysis efficiency and product quality (Figure 6a). For example, the surface of the liquid Ga cathode should be remained in the liquid state during electrolysis process in this work, aiming at maintaining selective reduction and UPD effect of liquid Ga cathode. In this process, real‐time evolution of Ti dendrites on the surface of the Ga cathode could not be observed or monitored via traditional empirical methods. After a long‐term electrolysis without in situ monitoring, the surface of the Ga cathode would be covered by solid Ti dendrities. Thus, the selective reduction would fail and the UPD effect of Ga cathode would be varied, which would impact the product quality using liquid cathode.

On the contrary, the novel in situ 4D visualization method reported here allows the “black box” for opening to a visible state (Figure 6b). In this way, real‐time and 3D electrode process would be monitored, by which the unknown evolution process of the electrode could be understood. These new findings promise a platform for directly and deeply understanding the electrode kinetics under multi‐physical fields, rather than analyzing the electrode dynamics via repeated ex situ results. Meanwhile, fundamental theory of electrochemistry under extreme conditions could be developed. More importantly, the process regulation of the electrolysis could be in situ confirmed, which is more efficient for improving the quality of electrodeposition products. Apparently, such 4D visualization strategy is a universal technology, and it can be widely employed into other electrochemical cells, specifically the opaque systems under extreme conditions. On the other hand, Wang et al.**^[^** ^43^, ^44^, ^45^, ^46^ **^]^** have successfully revealed the kinetic processes in the metal ion battery electrodes via X‐ray computer tomography techniques, which is favorable to advance battery science and technology. Apparently, the 4D techniques in the microscale could be used to well guide the 4D study of industrial electrodeposition, and the scientific gap between the macroscale and microscale should be well addressed.

## 4 Conclusion

In summary, X‐ray tomography was employed to demonstrate the 4D visualization of Ti electrodeposition in the corrosive ion liquid electrolyte. With the process of electrodeposition using both solid Ni and liquid Ga electrodes, quantitative analysis on morphologies, current efficiency, electric filed, concentration gradient was performed. To understand the unusual phenomenon of Ti electrodeposition on Ni and Ga electrodes, the “growth model of diffusion‐induced local field enhancement” was proposed. The methodology of opening the “black box” here would be the powerful tool in situ tracking the evolution of electrodeposition that could not be well achieved from the traditional electrodeposition technology.

## 5 Experimental Section

### Materials and Chemicals

l‐methyl‐3‐butyl‐imidazolium bis (trifluoro methyl sulfone) ([BMIm]BTA, 99%) was purchased from Tokyo Chemical Industry. Liquid gallium (99.9%) and anhydrous titanium tetrachloride (TiCl_4_, 99.9%) were purchased from Alfa Aesar. Ti wire (99.9%, 1 mm in diameter), Ni wire (99%, 1 mm in diameter), and Pt wire (99%, 0.5 mm in diameter) as well as Mo wire (99%, 0.5 mm in diameter) were received from Aladdin. All the metallic wires were polished by gauze, and cleaned by anhydrous alcohol prior to the experiments. The [BMIm]BTA was dried under vacuum at 120 °C for 24 h before use. A nominal 0.24 m TiCl_4_ ionic liquid was made by mixing TiCl_4_ and [BMIm]BTA in an argon atmosphere glove box (O_2_ and H_2_O less than 5 ppm).

### Electrolytic Cell

Electrolytic cell was made using a quartz tube (inner diameter: 10 mm) with a Teflon cap (Figure 1b, Figure S1d,e, Supporting Information). To find the optimal balance among the X‐ray tomography spatial resolution, operability, and practical value, the outside diameter of the quartz tube was set in 14 mm, and its corresponding inside diameter was 10–12 mm, which could provide about 11 µm spatial resolution that is acceptable for revealing a mesoscale kinetic process and also sufficient in terms of electrometallurgy or electroplating. Moreover, it is worth noting that, in situ X‐ray tomography technique does not need optical transparent electrolytic cell, however, for operating easily, quartz tube was chosen as a reaction tank. A Pt wire was selected as a quasi‐reference (Pt Quasi‐RE) electrode and a Ti wire was used as a counter electrode. In order to demonstrate the Ti electrodeposition at solid and liquid electrodes, respectively, both Ni wire and liquid Ga were used as working electrodes. The detailed manufacture process on the liquid Ga electrode was given in our previous works, while its schematically illustration and photograph are shown in Figure 1c and Figure S1f, Supporting Information, respectively.

### Electrochemical Measurements

All the electrochemical measurements including open circuit potential, polarization curve, cyclic voltammetry (CV), and SWV as well as potentiostatic electrodeposition were performed in abovementioned electrolytic cell with CHI electrochemical work station (660E, Shanghai Chenhua Instrument Co., Ltd.) at room temperature. Polarization curve was conducted at 10 mV s^−1^. CV measurement was carried out within the various voltage ranges obtained from the results of polarization curve, and scan rate was ranging from 10 to 30 mV s^−1^. Potentiostatic electrodeposition was performed at a potential of −2.2 V versus Pt Quasi‐RE. The electrodeposition time at Ni electrode and liquid Ga electrode was 960 and 720 min, respectively.

### In Situ X‐Ray Tomography

The assembled electrolytic cell was fixed vertically to the holder of microfocus X‐ray system (nanoVoxel‐4000, China Tianjin Sanying Precision Instrument Co., Ltd.), as shown in Figure 1a and Figure S1a–c, Supporting Information. The scans were carried out with X‐ray at 30 kV and a current of 0.5 mA. The scan time was about 30 min, which was indeed too long for an electrochemistry. However, it is worth noting that there are some associate technologies reported to deal with the data obtained by CT with high scan rate. For instance, Yang et al. have employed deep learning to deal with computed tomography images collected under a high scan rate and obtained good results.^[^ ^46^ ^]^ Obviously, we can reduce the scan time by using this associate technology. The spatial resolution is about 6–10 µm in this work. The 3D data was collected in 500–2000 slices, and each slice was separated by one spatial resolution. Subsequently, the final 3D results were reconstructed using Avizo software and, based on which, the quantitative data was achieved. The first 3D result was received by scanning the original electrode without electrodeposition, and then, the second 3D results was obtained by scanning the electrode after electrodeposition for 30 min. As an analogy, we eventually got the evolution of 3D morphology of the metallic Ti product with electrodeposition process time. The interval for each time X‐ray scan is determined by the electrodeposition rate. Because the metallic Ti deposition rate is low in ionic liquid, the interval was set from 30 to 510 min. Because the local field intensity around the Ni electrode during electrodeposition process was different, the average thickness (Δ*d*) of Ti plating at the Ni electrode was calculated statistically according to the follow equation:

(1) $$\Delta d\;=\frac{1}{2}\left[\frac{\left(\varphi_{h}^{i}-\varphi_{h}^{o}\right)}{2}+\frac{\left(\varphi_{v}^{i}{-\varphi }_{v}^{o}\right)}{2}\right]$$

where $\varphi_{h}^{o}$ and $\varphi_{v}^{o}$ are the horizontal diameter and vertical diameter of the original Ni electrode, respectively, and $\varphi_{h}^{i}$ and $\varphi_{v}^{i}$ represent the horizontal diameter and vertical diameter of the Ni electrode after Ti deposition. The schematically illustration for above calculation is shown in Figure S7, Supporting Information.

### Materials Characterizations

The Ni electrode after 4D electrodeposition for 960 min was taken out from the electrolytic cell and washed by using of anhydrous ethanol to remove residual electrolyte. The liquid Ga electrode after in situ electrodeposition was taken out from the electrolyte and placed more than 240 min, after which the whole Ga electrode was in liquid state. Subsequently, the liquid Ga was collected by using an injector. The collected Ni electrode and liquid Ga were analyzed by SEM equipped with an EDS probe (Thermo NS7).

### Density Functional Theory Calculations

DFT was carried out in the generalized gradient approximation with the Perdew–Burke–Ernzerhof functional for exchange and correlation effects of the electrons, as implemented in the DMol^3^ package. The convergence tolerance of energy was 1 × 10^−5^ Ha and the Brillouin‐zone integration was sampled by 3 × 3 × 1 special k‐points for atomic structure relaxation. The geometry optimization was set with the displacement and max‐force as 0.005 Å and 0.002 Ha Å^−1^. The binding energy (*G*_BE_) was defined as:

(2) $$G_{\mathrm{BE}}=G_{\mathrm{Ti}-X}-G_{\mathrm{Ti}}-G_{X}$$

where *G*_Ti_ and *G*_X_ are the ground state energy of the titanium and cathode matrix, *G*_Ti‐X_ is the total energy of titanium deposited on the matrix.

### Modelling and Simulation

The modelling and simulation were performed by using a COMSOL Multiphysics simulation. The physics model “tertiary current distribution and Nernst‐Planck(tcd)” was used. The Nernst‐Planck equation in the simulation is shown as follows:

(3) $$J_{i}=-D_{i}\nabla c_{i}-z_{i}u_{m,i}Fc_{i}\nabla \varphi_{i}$$

(4) $$\Sigma_{i}z_{i}\;c_{i}=0$$

(5) $$u_{m,i}=\frac{D_{i}}{RT}$$

where *D*_i_ is the diffusion coefficient, *J*_i_ the reaction flux of the species, *c_i_* the concentration of reactive ions, *F* the Faraday constant, *z*_i_ the valence, *u*_m,I_ the mobility, and *Φ*_i_ the electrolyte potential.

The simulation electrolytic cell (as shown in Figure 1b,c) consists of electrolyte with a diameter of 10 mm, Ni working electrode, Ti counter electrode. The balance potential of Ni electrode and Ti electrode were installed as −2.2 and −1.2 V (vs Pt Quasi‐RE) initially. The balanced current density of anode and cathode were 0.145 A m^−2^. The diffusion coefficient of Ti ions in the electrolyte was set to 4.4 × 10^−10^ cm^2^ s^−1^. The initial concentration of TiCl_4_ was 0.24 mol L^−1^. All above parameters were from experimental results. Other initial boundary conditions are obtained by coupling of COMSOL's modules.

The boundary conditions for the anode and cathode electrodes were given by the Butler‐Volmer equation for Ti electrodeposition. The electrodeposition process was assumed to take place through the following mechanism: Ti^4+^+e^−^ = Ti^3+^ and Ti^3+^+3e^−^ = Ti. Kinetic equation of electrode reaction on electrode surface was chosen “Concentration dependent kinetics.” Parameter settings used experimental results. The equation was written as follows:

(6) $$i_{\mathrm{loc}}=i_{0}\;(C_{O}e^{\left(\frac{\alpha_{a}F\eta }{RT}\right)}-C_{R}e^{\left(\frac{-\alpha_{c}F\eta }{RT}\right)}$$

where *i*_0_ is the exchange current density, *F* is the Faraday constant, *C*_i_ is the concentration, *α* is the transfer coefficient, *η* is the over‐potential.

(7) $$C_{O}=\frac{k_{1}}{k_{2}}\;C_{Ti^{3+\;}}$$

The reactions on the cathode can be simplified as follow:

(8) $$Ti^{4+}+{e^{-}}_{\underset{k_{2}}{\leftarrow }}^{\overset{k_{1}}{\to }}Ti^{3+}$$

(9) $$Ti^{3+}+3{e^{-}}_{\underset{k_{2}}{\leftarrow }}^{\overset{k_{1}}{\to }}\mathrm{Ti}$$

The overall reaction equation at the cathode could be simplified as follow:

(10) $$Ti^{4+}+4{e^{-}}_{\underset{k_{2}}{\leftarrow }}^{\overset{k_{1}}{\to }}\mathrm{Ti}$$

Compared to the reaction of $Ti^{3+}+3{e^{-}}_{\underset{k_{2}}{\leftarrow }}^{\overset{k_{1}}{\to }}\mathrm{Ti}$, if the reaction of $Ti^{4+}+{e^{-}}_{\underset{k_{2}}{\leftarrow }}^{\overset{k_{1}}{\to }}Ti^{3+}$ is so facile that it is always at equilibrium. So, there is an equation $k_{1}C_{Ti^{4+}}=k_{2}C_{Ti^{3+}}$.

The Nernst equation of the reaction of $Ti^{3+}+3{e^{-}}_{\underset{k_{2}}{\leftarrow }}^{\overset{k_{1}}{\to }}\mathrm{Ti}$ is

(11) $$E=E^{\theta }\;+\;\frac{RT}{nF}\;\ln\frac{C_{O}}{C_{R}}=E^{\theta }\;+\;\frac{RT}{nF}\;\ln\frac{C_{Ti^{3+}}}{C_{\mathrm{Ti}}}=E^{\theta }\;+\;\frac{RT}{nF}\mathrm{lnT}i^{3+}$$

The net reaction rate is

(12) $$v_{\mathrm{net}}=k_{f}C_{Ti^{3+}}-k_{b}C_{\mathrm{Ti}}=i/nFA$$

Now consider the special case in which the interface is at equilibrium with a solution. In this situation, *E*  =*E* ^*θ*^  and $C_{Ti^{3+}}=C_{\mathrm{Ti}}\;$, so that $k_{f}C_{Ti^{3+}}=k_{b}C_{\mathrm{Ti}}\;$ and *k*_f_ = *k*_b_ .

The forward rate constant and reverse rate constant have the same value *k*_0_, so *k*_0f_ = *k*_0b_ =  *k*_0_.

(13) $$k_{f}=k_{0}e^{-\alpha f(E-E^{\theta })}$$

(14) $$k_{b}=k_{0}e^{(1-\alpha )f(E-E^{\theta })}$$

(15) f=nF/RT

Insertion of these relations into the equation of $v_{\mathrm{net}}=k_{f}\;C_{Ti^{3+}}\;-k_{b}C_{\mathrm{Ti}}=i/nFA$ yields the complete current‐potential characteristic:

(16) $$i=nFAk_{0}\left[C_{Ti^{3+}}e^{-\alpha f(E-E^{\theta })}-C_{\mathrm{Ti}}e^{(1-\alpha )f(E-E^{\theta })}\right]$$

Insertion of the equation of $k_{1}C_{Ti^{4+}}=k_{2}C_{Ti^{3+}}$ into the above equation, the relation between over‐potential and current density can be written:

(17) $$i=nFAk_{0}\left[k_{1}/k_{2}C_{Ti^{4+}}e^{-\alpha f(E-E^{\theta })}-C_{\mathrm{Ti}}e^{(1-\alpha )f(E-E^{\theta })}\right]$$

According to above description, the electrodeposition rate of Ti on the Ni electrode was given in the software. The grid deformation of the model at the cathode was described in real time, which represent the 3D morphology evolution.

(18) $$R_{\mathrm{deposited}}=\frac{Mi_{\mathrm{loc}}}{\rho nF}$$

where *ρ* is the density of Ti and *M* is the molar mass.

In order to intuitively understand the growth of Ti nucleus on Ni and Ga electrodes, we performed the other two simulations. In these cases, the initial geometric model was established according to the results achieved from X‐ray tomography and the corresponding assumption. A 2D rectangle was selected to represent the local region near the interface between electrode and electrolyte. Top region was used as Ti counter electrode and the bottom region was employed as Ni or Ga working electrode. In addition, compared with the difference of the diffusion coefficient of Ti atom in metal electrode, the other differences between Ni and Ga are negligible. Hence, most of the parameters and boundary conditions were same on both Ni and Ga electrodes apart from the diffusion coefficient. A “dilute material diffusion(tds)” module was employed. Meanwhile, to simple the calculation process, Fick's first law was used in the transfer module of dilute matter, as shown follow:

(19) $$J_{i}=D_{i}\nabla c_{i}$$

where *J*_i_ is the molar flux (mol m^−2^ s^−1^), *C*_i_ the concentration (mol m^−3^), and *D*_i_ the diffusion coefficient (m^2^ s^−1^).

### Statistical Analysis

There is not special pre‐processing of the results data shown in this work except otherwise noted in the text. Results data were given as mean for three to five replicates for each experiment. Analysis on CT slices was conducted using Avizo 9.0 software.

## Conflict of Interest

The authors declare no conflict of interest.

## Supporting information

Supporting Information

Supplemental Movie 1

Supplemental Movie 2

Supplemental Movie 3

Supplemental Movie 4

Supplemental Movie 5

## Data Availability

Research data are not shared.
