# Supplementary material for: Quantificational 4D Visualization of Industrial Electrodeposition
Source: Adv Sci (Weinh). 2021 Oct 28;8(24):2101373. doi: 10.1002/advs.202101373 (PMC8693065; doi:10.1002/advs.202101373)
Supplement: Supplementary file 1 — Supporting Information [file ADVS-8-2101373-s006.pdf]

WILEY-VCH

© 2021 Wiley-VCH GmbH

ADVANCED SCIENCE
Open Access

Supporting Information

for *Adv. Sci.*, DOI: 10.1002/advs.202101373

Quantificational 4D visualization of industrial electrodeposition

Handong Jiao, Zhaoliang Qu, Shuqiang Jiao, Yang Gao, Shijie Li, Wei-Li Song,* Mingyong Wang, Haosen Chen, Daining Fang**

Supporting Information

Quantificational 4D visualization of industrial electrodeposition

Handong Jiao,† Zhaoliang Qu,† Shuqiang Jiao, Yang Gao, Shijie Li, Wei-Li Song,* Mingyong Wang, Haosen Chen, Daining Fang**

Dr. H. Jiao, Prof. Z. Qu, Prof. S. Jiao, S. Li, Prof. W.L. Song, Prof. H. Chen, Prof. D. Fang

Institute of Advanced Structure Technology, Beijing Institute of Technology, Beijing 100081, P. R. China.

E-mail: sjiao@ustb.edu.cn; weilis@bit.edu.cn; fangdn@bit.edu.cn

Y. Gao, Prof. S. Jiao, Prof. M. Wang

State Key Laboratory of Advanced Metallurgy, University of Science and Technology Beijing, Beijing 100083, P. R. China.

[b] State Key Laboratory of Advanced Metallurgy, University of Science and Technology Beijing, Beijing, 100083, P R China

† These authors made equal contribution to this work.

Figure S1. Photographic images of the 4D characterization device. (a) Side view of the assembled 4D characterization system. (b and c) Front view and side view of the spatial location-relation between the X-ray source and the electrolysis cell. (d and e) Photographic images of the electrolysis cells configuring with a solid Ni working electrode and a liquid Ga working electrode. (f) Photographic image of the liquid Ga working electrode.

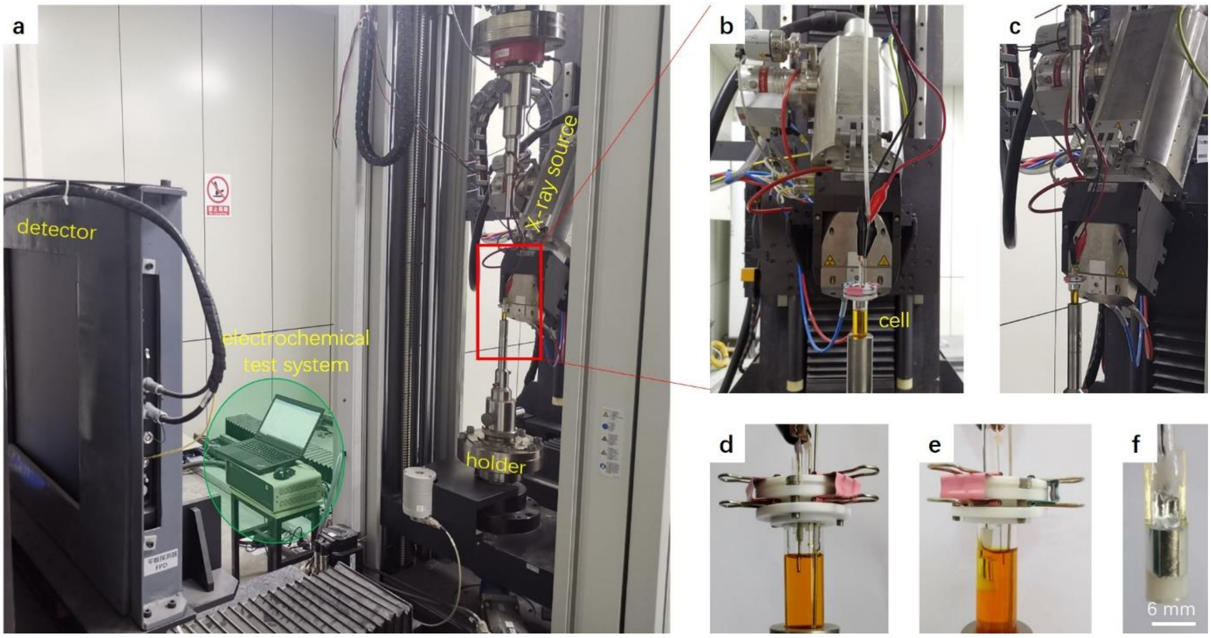

Figure S2. The stability evaluation of Pt quasi reference electrode. Open circuit potential curves of Ni working electrode and Ga working electrode with time.

The OCP curves in the both electrode systems assembled with Ni or Ga working electrode demonstrate a narrow fluctuated potential range at the initial stages, followed by a steady value. The results indicate the validity of using the Pt Quasi-RE in the electrolytic cell.

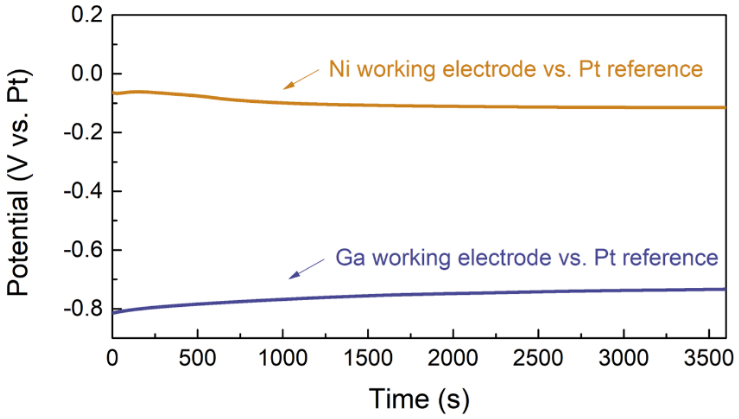

Figure S3. The measurement of the electrochemical window of the blank electrolyte on two types working electrodes. (a) Cathodic and (b) anodic polarization curves of Ni working electrode in [BMIm]BTA ionic liquid. (c) Cathodic and (d) anodic polarization curves of Ga working electrode in [BMIm]BTA ionic liquid.

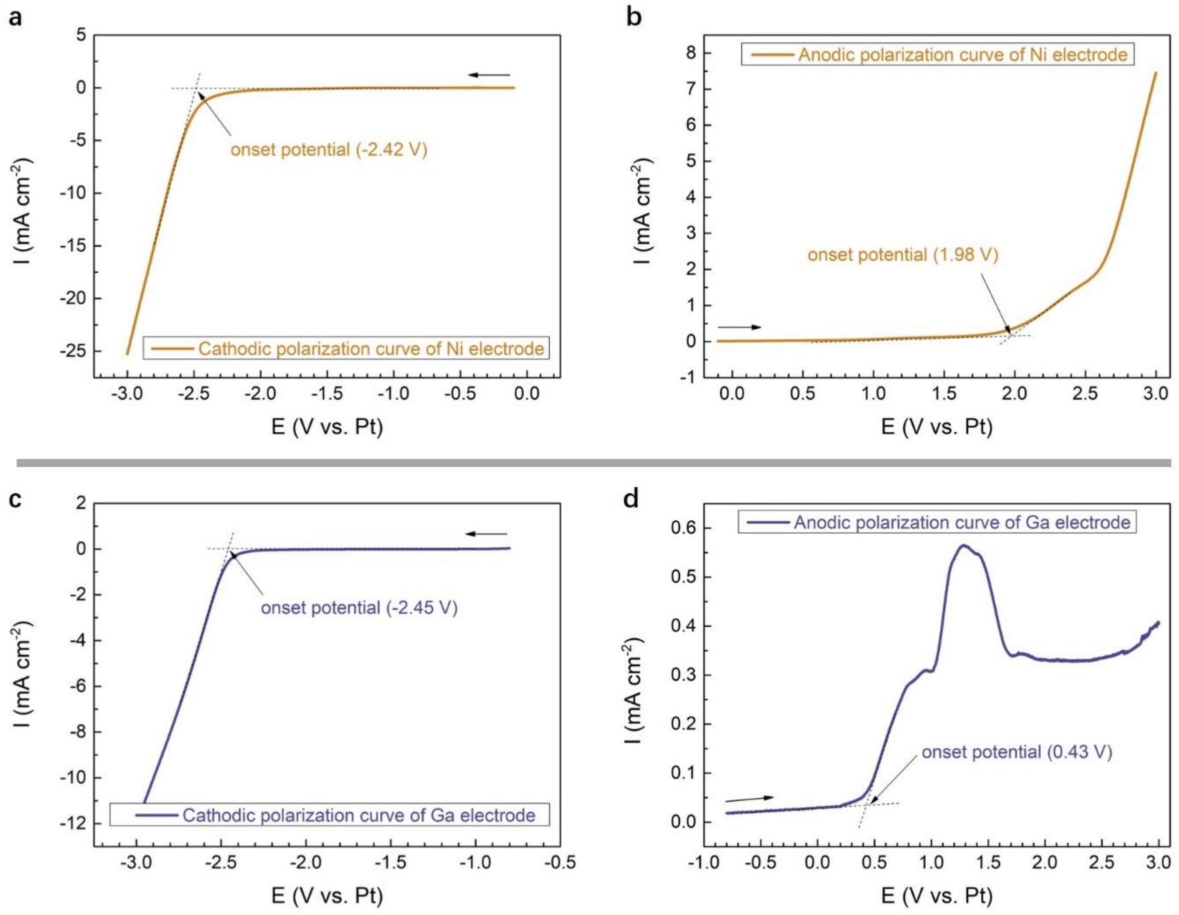

Figure S4. The extended electrochemical measurements of Ti atom on Ni and Ga electrodes. Cyclic voltammograms of Ti on (a) Ni and (b) Ga working electrode. (c) The magnification plot of the CV of (b). (d) The polarization value (Δ*U*) under different scan rates. Schematic mechanisms of (e) the formation of the Ti monoatomic layer on solid Ni substate and (f) the diffusion of metallic Ti atom into liquid Ga substate during UPD process.

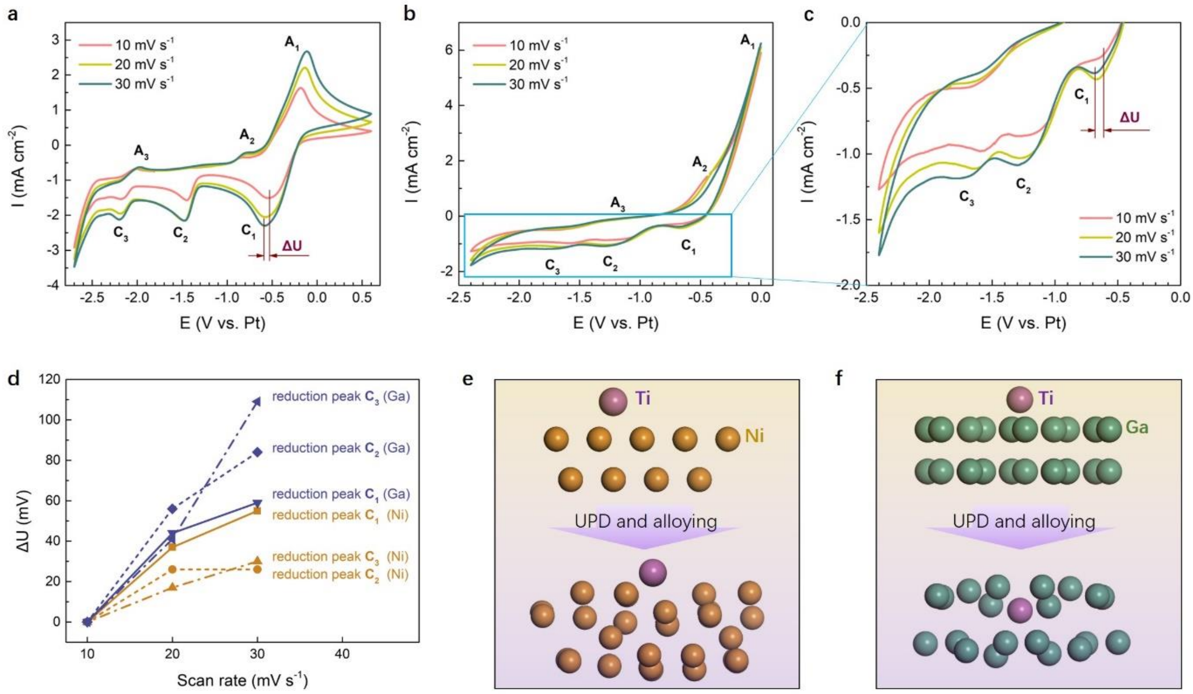

Figure S5. The current-time profile of Ti electrodeposition on the Ni electrode. Potential: -2.2 V vs. Pt Quasi-RE.

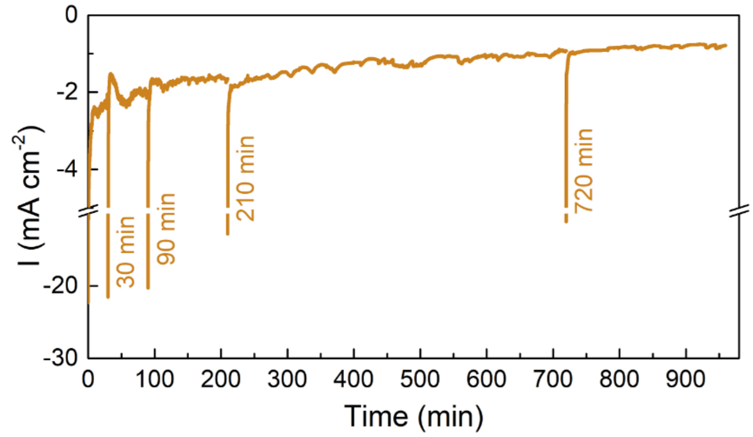

Figure S6. The distribution of Ti atom on the Ni surface. (a) SEM image of the Ni surface after Ti electrodeposition for 960 min. (b) Ni and (c) Ti distribution on the Ni surface obtained by EDS test. (d) The mass percent of Ti on the Ni surface.

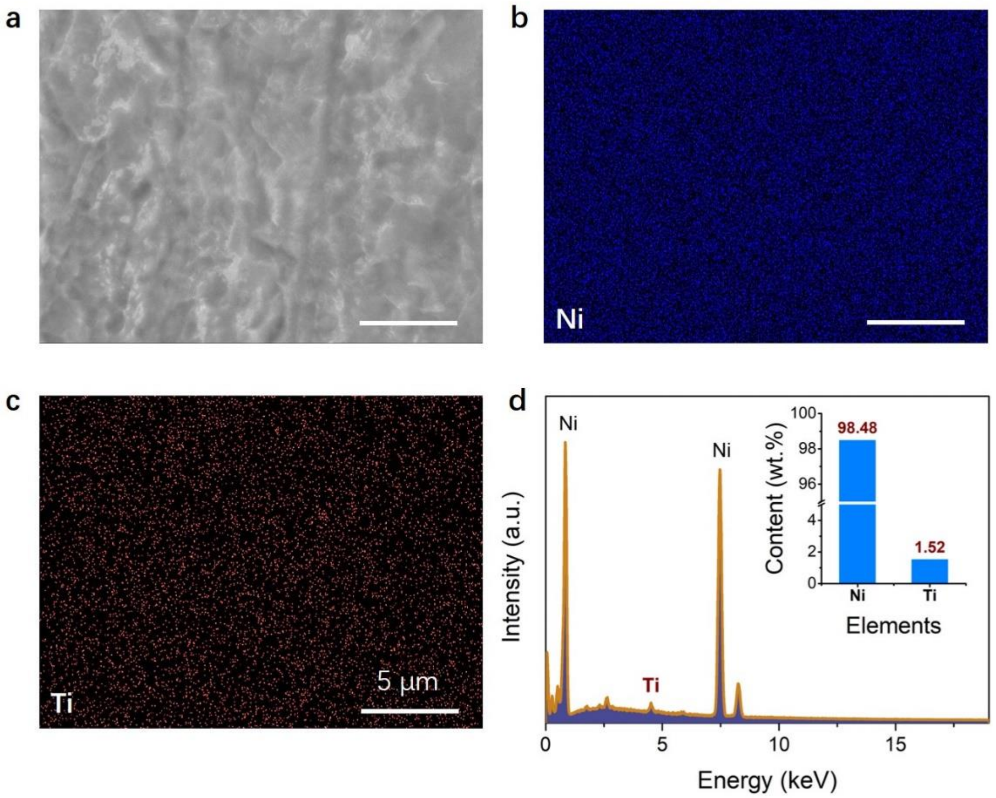

Figure S7. (a) The extended cross-section slices of the Ni electrode by X-ray tomography at different electrodeposition time. (b) The schematically illustration for the calculation of the average thickness of Ti plating.

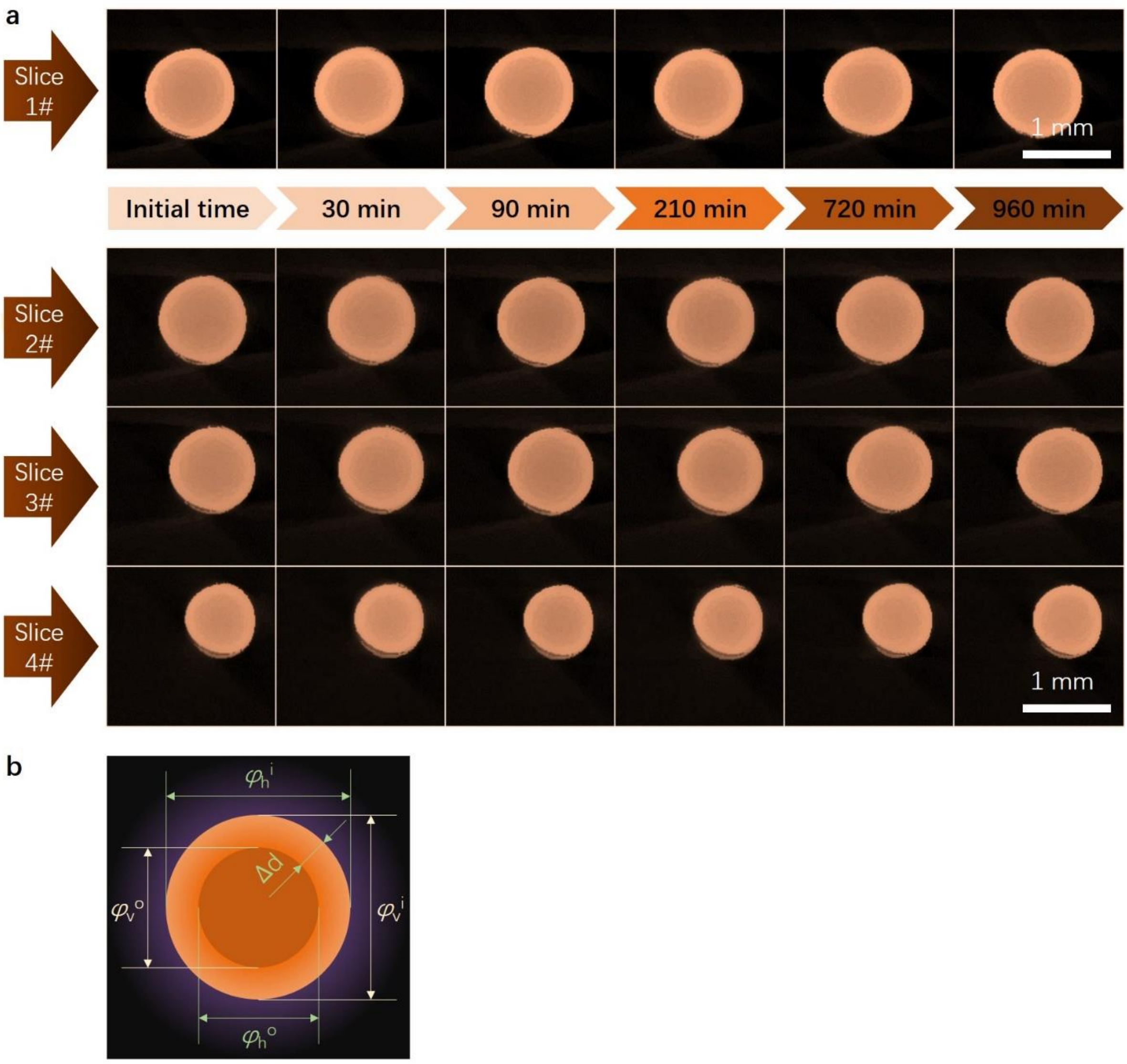

Figure S8. The microtopography characterization of the Ni and Ti electrodes after electrolysis. (a) SEM images of the Ni working electrode before (left) and after (right) electrolysis for 960 min, which indicates the roughness was about 2-3 μm. (b) SEM images of the Ti counter electrode before (left) and after (right) electrolysis for 960 min. (c) Schematic illustration of the mesoscale smooth surface and the microscopic rough morphology obtained by CT scan and SEM characterization, respectively.

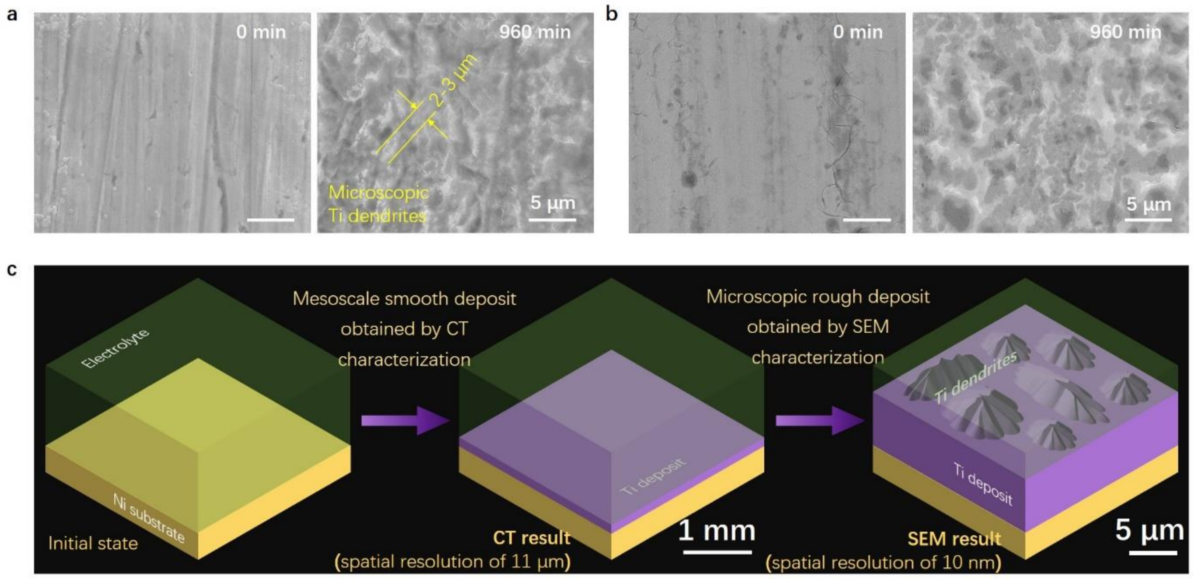

Figure S9. The evolution of the concentration distribution of $TiCl_4$ with time in the electrolytic cell. (a) 3D, (b) 2D vertical-section, and (c) 2D cross-section images of the change of the distribution of $TiCl_4$ with time in electrolytic cell.

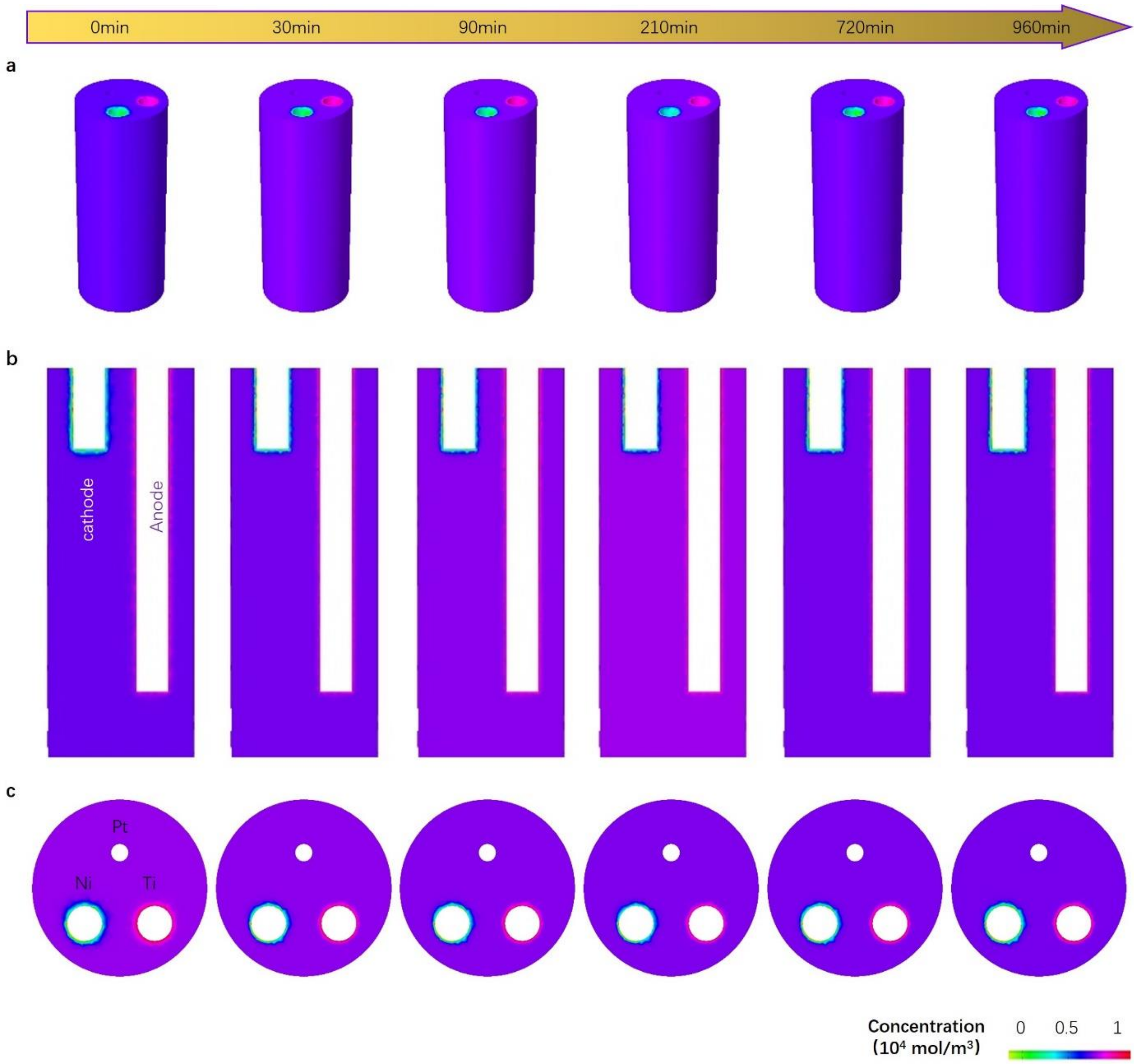

Figure S10. The evolution of the electric field with time in the electrolytic cell. (a) 3D and (b) 2D cross-section images of the change of the distribution of electric field with time.

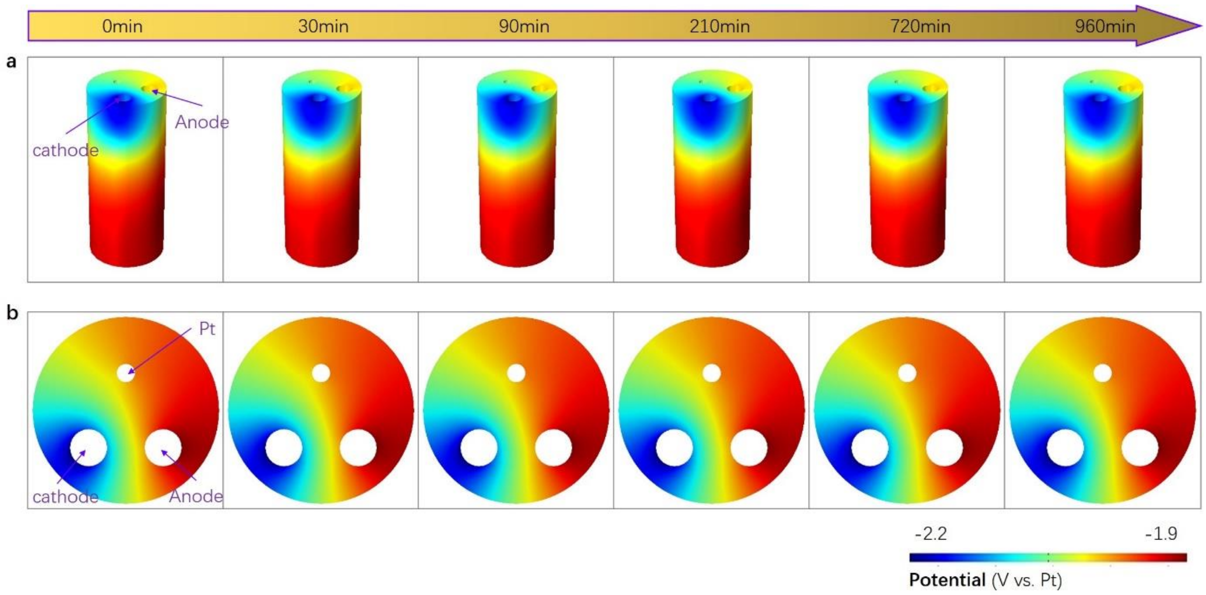

Figure S11. The evolution of the electric field with tim in the electrolytic cell. (a) 2D vertical-section and (b) cross-section images of the change of the distribution of current density with time. (c) Magnification images of the distribution of current density around of the cathode.

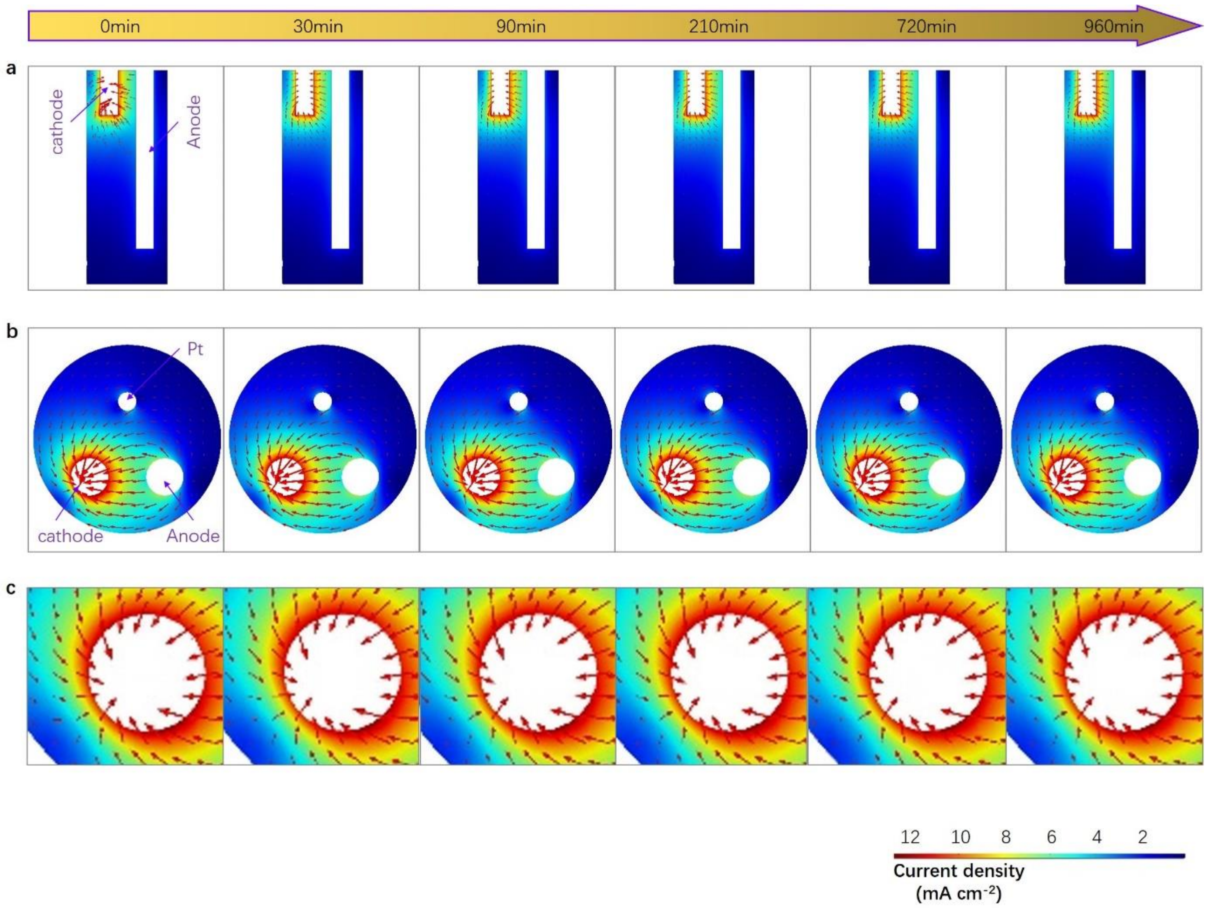

Figure S12. The changes of the thickness of Ti plating on Ni electrode and the dissolution on Ti electrode during electrolysis.

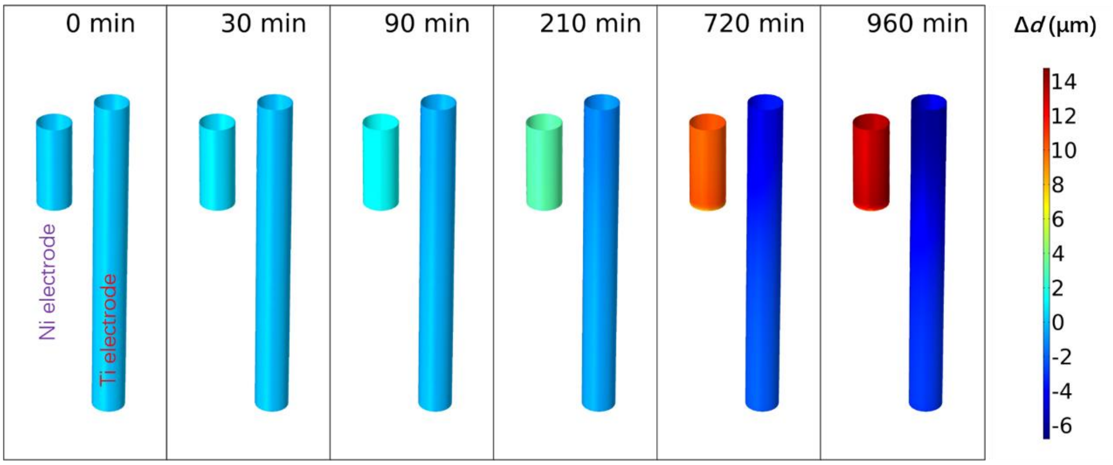

Figure S13. The current-time profile of Ti electrodeposition on the Ga electrode. Potential: -2.2 V vs. Pt Quasi-RE.

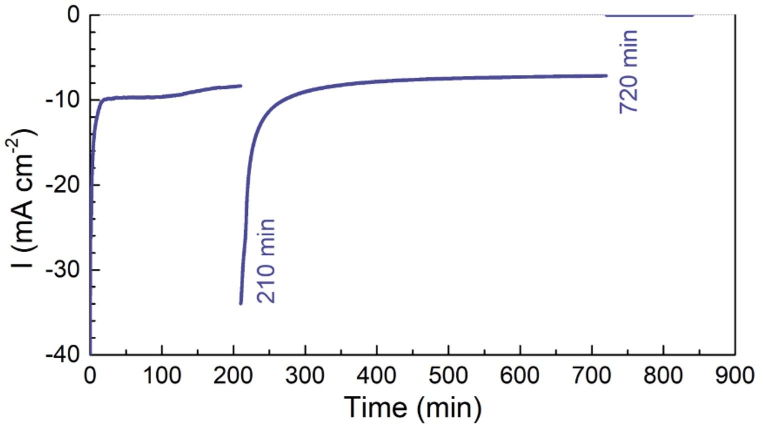

Figure S14. SEM images of the liquid Ga electrode after Ti electrodeposition for 720 min and placing for 240 min.

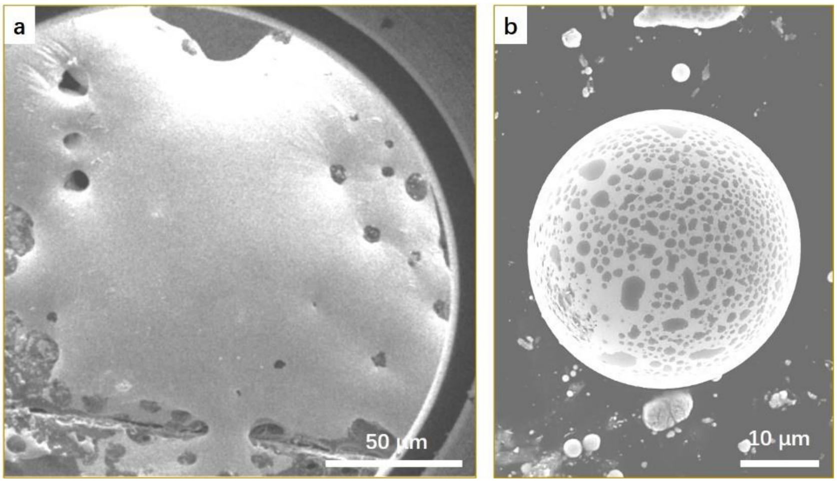

Movie S1.mpg

Movie. S1. The 3D rendering result of the electrolytic cell by X-ray tomography technique.

Movie S2 The evolution of the 3

Movie. S2. The evolution of the 3D concentration field of titanium tetrachloride with time.

Movie S3 The evolution of the 3

Movie. S3. The evolution of the 3D electric field.

Movie S4 The evolution of 2D ve

Movie. S4. The evolution of 2D vertical-section and cross-section of current density.

Movie S5 The changes of the th

Movie. S5. The changes of the thickness of Ti plating on Ni electrode and the dissolution on Ti electrode during electrolysis.
